# Crucial role of oxygen on the bulk and surface electronic properties of stable β phase of tungsten

**DOI:** 10.1038/s41598-022-07658-7

**Published:** 2022-03-09

**Authors:** Ananya Chattaraj, Sebastien Joulie, Virginie Serin, Alain Claverie, Vijay Kumar, Aloke Kanjilal

**Affiliations:** 1 Department of Physics, School of Natural Sciences, Shiv Nadar University, NH-91, Tehsil Dadri, Gautam Buddha Nagar, Uttar Pradesh 201 314 India; 2 CEMES-CNRS and Université de Toulouse, 29 rue J. Marvig, 31055 Toulouse, France; 3 Center for Informatics, School of Natural Sciences, Shiv Nadar University, NH91, Tehsil Dadri, Gautam Buddha Nagar, Uttar Pradesh 201 314 India; 4 Dr. Vijay Kumar Foundation, 1969 Sector 4, Gurgaon, Haryana 122001 India

**Keywords:** Electronic structure, Surfaces, interfaces and thin films, Electronic properties and materials

## Abstract

The A15 β phase of tungsten has recently attracted great interest for spintronic applications due to the finding of giant spin-Hall effect. As β phase is stabilized by oxygen, we have studied the electronic structure of O-doped β-W from first principles calculations. It is found that 20 at.% O-doping makes β phase lower in energy than α-W. These results are in good agreement with energy dispersive X-ray spectroscopy which also shows ~ 16.84 at.% O in 60 nm thick W films. The latter has predominantly β phase as confirmed by grazing incidence X-ray diffraction (XRD). The simulated XRD of bulk β having 15.79 at.% O also agrees with XRD results. Oxygen binds strongly on the surface and affects the Dirac fermion behavior in pure β-W. There is structural disorder, O-inhomogeneity, and higher density-of-states in O-doped β-W at E_F_ compared with pure α. These results are promising to understand the properties of β-W.

## Introduction

The growing interest in spintronic devices has led to much attention on β-W due to the finding^1^ of the largest spin-Hall angle (SHA) of around − 0.5 among the metallic elements as well as a large spin–orbit torque (SOT)^1–6^. Also, the temperature coefficient of resistivity is nearly zero that is attractive for such applications. Bulk tungsten usually exists in the body-centered cubic (bcc) structure, known as α phase^7^, but a metastable β phase with A15 structure has been found in thin films^8–12^. Pure β-W has a closed packed structure and interestingly shows multiple Dirac nodal lines and massive Dirac fermion behavior near the Fermi energy (E_F_)^13^. However, experiments show that β-W becomes stabilized in the presence of oxygen^1,9,10,12,14–18^. But an understanding of the electronic structure of O doped β-W is lacking. Here we present results of the electronic structure calculations of bulk β-W doped with O as well as interaction of oxygen on (001) surface of α- and β-W. It is found that the bands and Dirac fermion behavior of pure β-W are affected by O doping. Our calculations show that the doping of 20 at.% O makes β phase favorable over α and it supports the energy-dispersive X-ray spectroscopy (EDX) and grazing incidence X-ray diffraction (GIXRD) results.

Earlier, we elucidated the transition of α to β phase of W with the doping of 25–30 at.% O using density functional theory (DFT) based ab initio molecular dynamics (MD) simulations^18^. However, our GIXRD and EDX experiments showed the formation of the β phase in 60 nm thick W films with 13–19 at.% O, while a higher concentration (16–22 at.%) in 35 nm film led to the formation of a very disordered structure with small nanocrystallites^18^. Therefore, a lower concentration of oxygen seems to be enough for the formation of β-W. Also, Demasius et al.^1^ oxidized 4.4 nm to 10 nm thick W films by controlled exposure of oxygen. It was shown that the films were in α phase without O, while ~ 12.1 at.% O was present in the β films that had the highest SHA. They observed an enhancement in SOT by incorporating O in W films but it was not much dependent on the amount of O in the films. It was suggested that the increase in SOT was governed by the interface between β-W and FeCoB magnetic film. In a different study using Pt as a heavy metal instead of W, Qiu et al*.*^19^ have shown a change in SHA due to the oxidation of the magnetic film by the migrated O from MgO/SiO_x_ layers. Accordingly, oxygen in β-W can affect the properties of the thin magnetic film grown on it and the interface^2,3^. β-W also has a higher superconducting critical transition temperature T_c_ (1 to 4 K) compared with 0.01 K in α-W^20–23^. The higher T_c_ of β-W is promising for developing detectors for astronomical and quantum information applications^24,25^. According to the Bardeen-Cooper-Schrieffer (BCS) theory of superconductivity, the DOS at the E_F_ can affect T_c_^25^. These applications of β-W and its higher resistivity^2,9,11,26^ of 100–300 μΩ-cm compared with ~ 5.33 μΩ-cm for α-W at room temperature need a proper understanding of the crucial role of O in its stability as well as atomic and electronic structure. Moreover, Derunova et al*.*^27^ have shown a giant spin-Hall effect in a group of A15 materials and emphasized the location of E_F_ in the energy bands to be very important. In this direction the doping of Ta has been shown to even further increase SHA^27–29^. Here we study the effects of oxygen on the electronic structure of β-W.

## Results and discussion

### Atomic structure and oxygen composition from experiments and calculations

**Figure 1:**
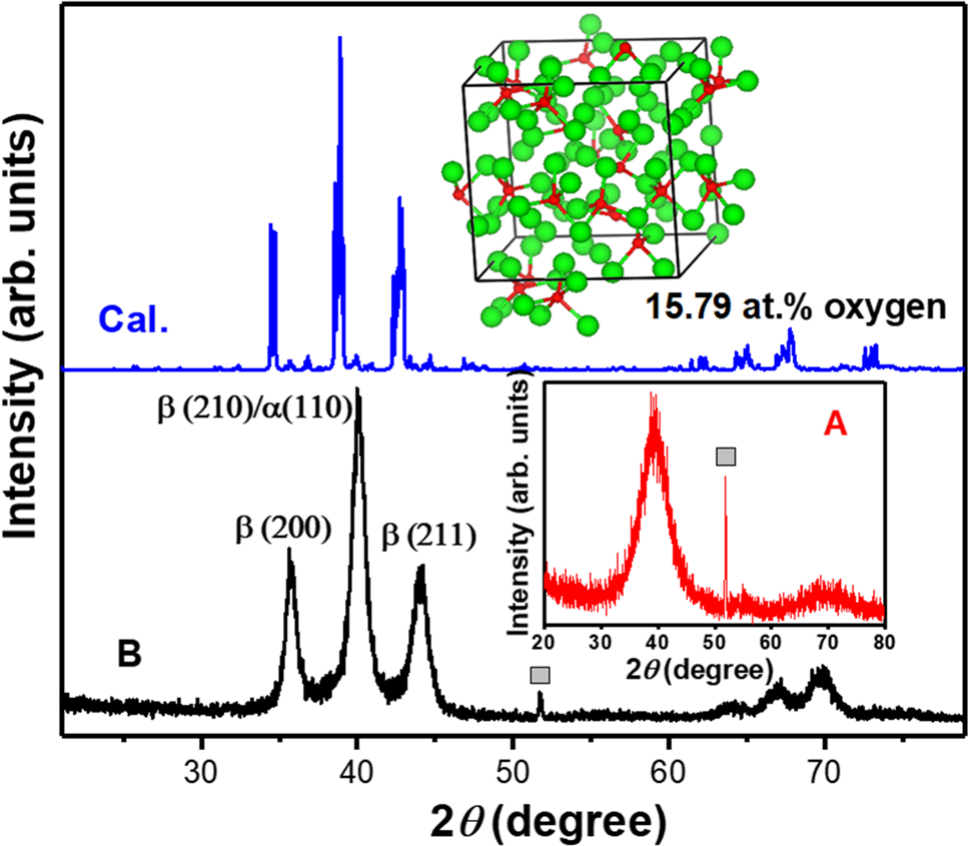
X-ray diffraction and atomic structure. X-ray diffraction pattern of film B (60 nm thick) with characteristic peaks of the β phase and possible presence of α phase due to the overlapping (110) peak. The calculated powder X-ray diffraction of 15.79 at. % O doped β-W obtained from *ab-initio* MD simulations reproduces the features quite well. The corresponding atomic structure is shown in the inset. The green and red balls are W and O atoms, respectively. The measured diffraction pattern of film A (35 nm thick) is shown in the inset. There is a broad peak signifying the presence of disorder in the structure. The peak marked with a grey box is the reflection from the Si substrate.

The GIXRD patterns of 35 nm (film A) and 60 nm (film B) thick W samples in the 2$\theta$ range of 20º to 80º are exhibited in Fig. 1 along with the calculated powder diffraction of a 2 × 2 × 2 supercell of β-W doped with 15.79 at.% O. Here $\theta$ is the Bragg angle. The atomic structure of O doped β-W was simulated using ab initio molecular dynamics (MD) at a high temperature of 3500 K in order to overcome barriers for O diffusion. The peak centred around 40º for film B belongs to the reflection from either (210) β-W or (110) α-W or both the phases (JCPDS #03–065-6453 for β-W, #04–0806 for α-W). The other two characteristic reflections from the (200) and (211) planes of the β phase are also identified in film B along with small peaks around 70º. Importantly, the same diffraction pattern was obtained almost a year ago suggesting the stability of the β-W phase. The diffraction pattern of the simulated O-doped β-W resembles the one obtained from GIXRD experiments on film B. The optimized O-doped β-W supercell (inset, Fig. 1) shows a slight deviation from a cube. There is a small increase in the lattice parameters to 10.32 Å, 10.36 Å, and 10.41 Å compared with the calculated value of 5.059 Å for the unit cell of pure β-W. There is some disorder in the structure and clustering of O atoms. In our earlier work^18^ we calculated the solution energy defined as E_S_ = E_NWO_ – E_NW_ – μ(O) for the doping of O in α and β phases. Here E_NWO_ is the total energy of the supercell containing N atoms of W and one O atom, E_NW_, the energy of the supercell of W with N atoms, and µ(O), the chemical potential of oxygen which is taken as the binding energy of O in an O_2_ molecule. The calculated results showed tetrahedral interstitial site to be the most favorable one for O doping in both the phases. Importantly the local environment of O in α-W becomes similar to the one in β-W and there is a large gain of 1.188 eV in the solution energy in β-W over the value in α-W. This is responsible for the phase transition from α to β in W with O-doping. Furthermore, there is attractive interaction between two O atoms in both the phases which leads to a further gain of 0.339 (1.417) eV with the O–O distance of 2.431 (2.285) Å in β-(α-)W. This leads to oxygen inhomegeneity with some regions having no O atom in the simulated structure. It should be noted that in some experiments, formation of β phase has been reported at lower oxygen concentrations. In such cases, the samples are likely to have a mixture of both α and β phases.

Our calculated diffraction peaks are slightly shifted to lower 2$\theta$ values compared with the experimental results. This is due to the slight overestimation of the calculated lattice parameters of pure α (3.171 Å) and β (5.059 Å) phases compared with the experimental values of 3.16 Å and 5.04 Å, respectively. Another possible reason could be the variation in the O concentration in the sample. In general, an increase in O concentration leads to an increase in the lattice parameters. Our EDX experiments suggest that oxygen concentration of about 15 at.% on an average is good to achieve a stabilized polycrystalline β-W film as discussed below. With this O concentration, all the W atoms in a sample may not be in the β phase due to O clustering as discussed above. Therefore, our results suggest the possibility of the co-existence of α phase with low O concentration together with β-W with much higher O concentration. A coexistence of α and β phases was also reported by Petroff et al.^9^ using transmission electron microscopy (TEM). We also performed simulated annealing calculations with ab initio MD on a 2 × 2 × 2 supercell of β-W and 4 × 4 × 4 supercell of α-W with the same O concentration. It is found that the doping of 20 at.% O makes the optimized β-W phase lower in energy compared with α-W. Therefore, a transition to β phase must occur by this O concentration. This is in good agreement with our experimental results.

**Figure 2:**
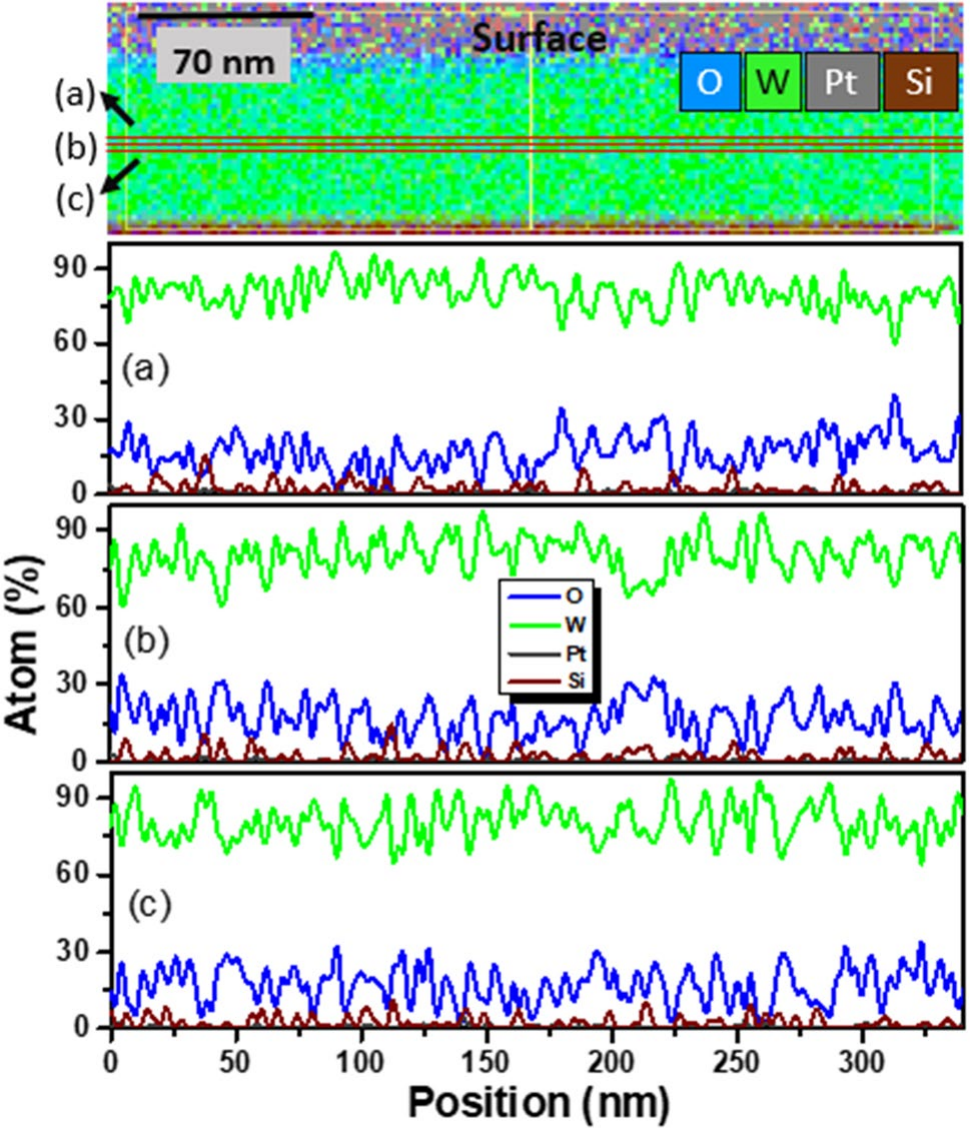
Elemental distribution in 60 nm thick film by EDX. The superimposed elemental EDX maps show the distribution of W (green), Si (brown), O (blue) and Pt (gray) in cross-sectional mode. It reveals the existence of O throughout the W film, but there is an increase in O concentration at the surface (see Fig. S2). The elemental profiles along the traces, marked by 3 red lines (**a**), (**b**) and (**c**) in the middle of the W film (top panel), are projected in the below panels showing the average oxygen concentration of (**a**) ~ 16.76 at.%, (**b**) 16.93 at.%, and (**c**) 16.84 at.% with the standard deviation of 9.38 at.%, 9.79 at.%, and 10.31 at.%, respectively, showing a significant variation on the averall trend. The Si and Pt signals within the W film are below the noise level.

We further performed EDX measurements in cross-sectional TEM (XTEM) mode parallel to the film surface to understand the distribution of the constituent elements. Figure 2 shows scans of the elemental compositions of W, O, Si and Pt in three regions (a–c) parallel to the surface in the middle of the film B. The superimposition of the EDX maps of W, O, Si and Pt is shown in the top panel. It is seen that the average oxygen concentration is (a) 16.76 at.%, (b) 16.93 at.%, and (c) 16.84 at.% with the standard deviation of 9.38 at.%, 9.79 at.%, and 10.31 at.%, respectively. This agrees with our earlier results (Ref. 19) of 13–19 at.% O from scans across the film. However, a close inspection of the EDX map indicates accumulation of O at the W surface. This could happen due to the reaction of oxygen with the surface W atoms because of exposure to ambient atmosphere for more than a month prior to the preparation of the XTEM sample. The 172 × 46 pixels in the EDX hypermaps with a spot size of 2 nm show that the variation of O concentration is almost similar in the bulk region as obtained from the traces a-c. However, oxygen concentration is comparatively higher (mean value 41.48 at.%) near the W surface (see Fig. S2 in Supplementary Information).

The EDX results of film A for 69 × 28 pixels are shown in Fig. S3 in the Supplementary Information. It shows higher mean O concentration (17.75 at.%) compared with the film B. The GIXRD measurements on film A (35 nm thick) further show a broad peak centred at around 2$\theta$ = 40º (inset, Fig. 1) suggesting it to be disordered. This is in agreement with the results of our ab initio MD simulations on higher O concentrations (20 at.%, ~ 27 at.% and ~ 30 at.%) in β-W. The calculated powder diffraction patterns show an increase in disorder with the broadening of the peaks as demonstrated in Fig. S1 in Supplementary Information. There is a corresponding slight increase in the lattice parameters and energy gain due to O doping and the values are listed in Table S1 in Supplementary Information.

### Electronic density of states of bulk phases with O doping

**Figure 3:**
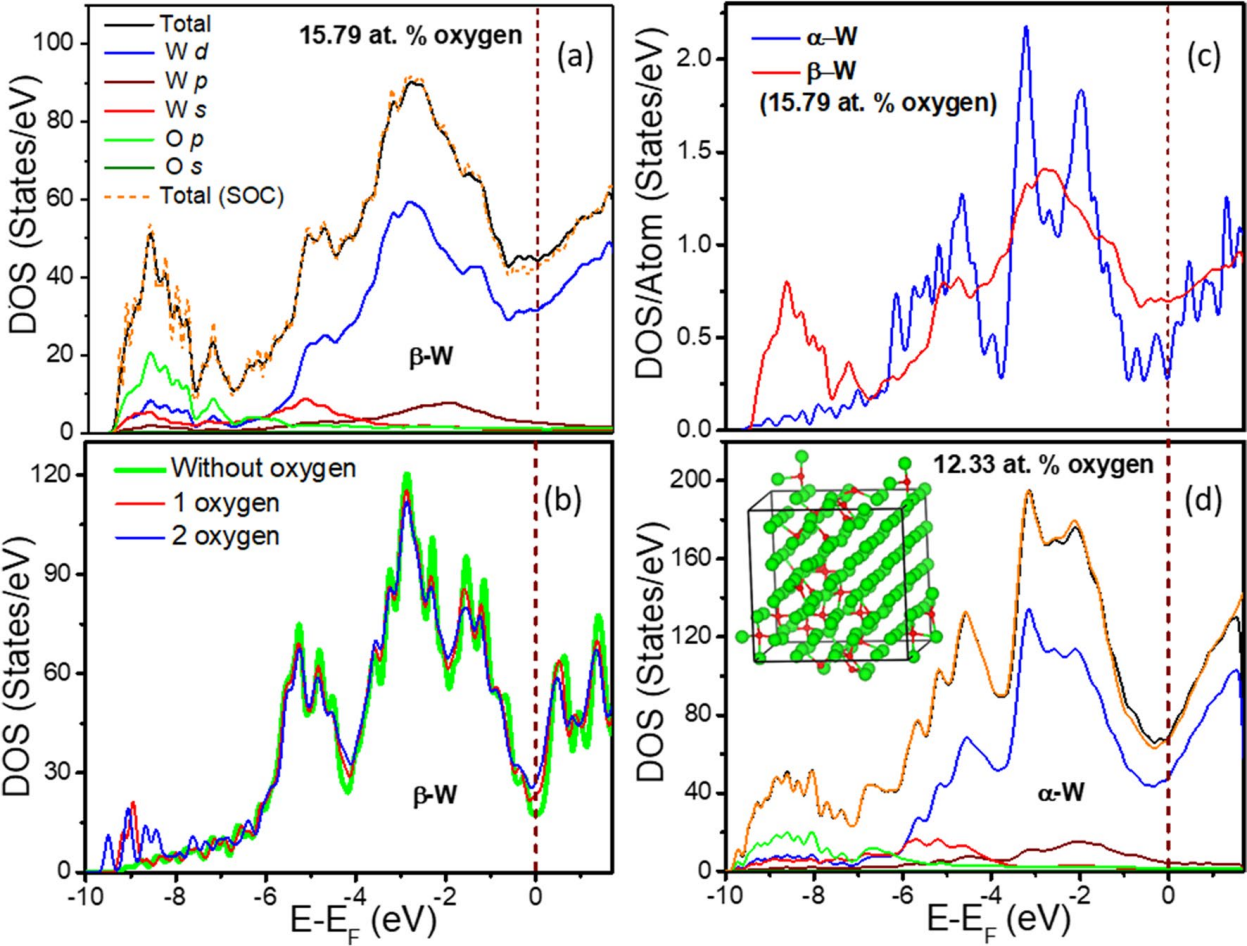
Electronic structure of bulk α- and β-W with O doping and spin–orbit coupling. (**a**) The total density of states (TDOS) with and without spin–orbit coupling (SOC) for a 2 × 2 × 2 supercell of β-W doped with 15.79 at.% O. Site and angular momentum projected density of states (PDOS) are shown without SOC. (**b**) The calculated DOS of a pure 2 × 2 × 2 supercell of β-W, and with one and two oxygen doped in it, shows an increase in the DOS at the Fermi level with the number of dopant. (**c**) The DOS per W atom for 15.79 at.% O doped β-W shows a significant enhancement at E_F_ compared with the value for pure α-W. (**d**) The TDOS with and without spin–orbit coupling for 4 × 4 × 4 supercell of α-W doped with 12.33 at.% O. The site and angular momentum projected DOS are also shown. The optimized atomic structure of α-W (green balls) showing clustering of oxygen (red balls) is given in the inset. The wine color dashed line shows the Fermi level at 0 eV in all the cases.

The calculated DOS of β-W with 15.79 at.% O is shown in Fig. 3. There is a broad peak at ~ 2.65 eV with a shoulder at ~ 1.5 eV below E_F_. There is also a weak peak at ~ 5 eV below E_F_. The angular momentum as well as site decomposed partial DOS (PDOS) show that the states appearing in the range of 0 to 6 eV arise mainly from the W 5*d* orbitals [Fig. 3a] and are also present in pure α-W. However, in the latter case there are two peaks at ~ 2 eV and ~ 3 eV below E_F_. Our results on pure α-W [Fig. 3c] agree well with those obtained by Jansen and Freeman^30^. The states beyond ~ 5 eV below E_F_ arise from the hybridization of O 2*p* with W 5*d* orbitals. Figure 3b further indicates an increase in DOS at E_F_ with increasing O concentration. Interestingly, the DOS at E_F_ is larger for 15.79 at.% O doped β-W compared with the pure α case [see Fig. 3c]. This is interesting for the understanding of higher T_c_ in β-W films within the BCS formalism but other factors such as the electron–phonon interaction, the microstructure and defects need also to be considered. Figure 3a shows the DOS for β-W with ~ 15.79 at.% O while Fig. 3d shows it for a simulated bulk α-W with ~ 12.3 at.% O. Interestingly, both show broadly similar features, although the atomic structures are different. The inset of Fig. 3d also shows the structure. It is found that even in the simulated and optimized α phase with lower O concentration, there is a clustering of oxygen due to attractive interaction as it could be expected. Both the O doped phases show a bonding peak due to oxygen at ~ 7 eV below E_F_. Furthermore, our calculations for O-doped bulk β-W also give a peak (Fig. 3) in the region of 8.6–9.4 eV below E_F_, while for the O-doped bulk α-phase the peak extends to slightly higher binding energies. These peaks are also identified to arise from the hybridization of O and W valence orbitals. The inclusion of spin–orbit coupling (SOC) does not change the DOS significantly except for a small decrease at E_F_.

### Electronic density of states of (001) surface and O adsorption

**Figure 4:**
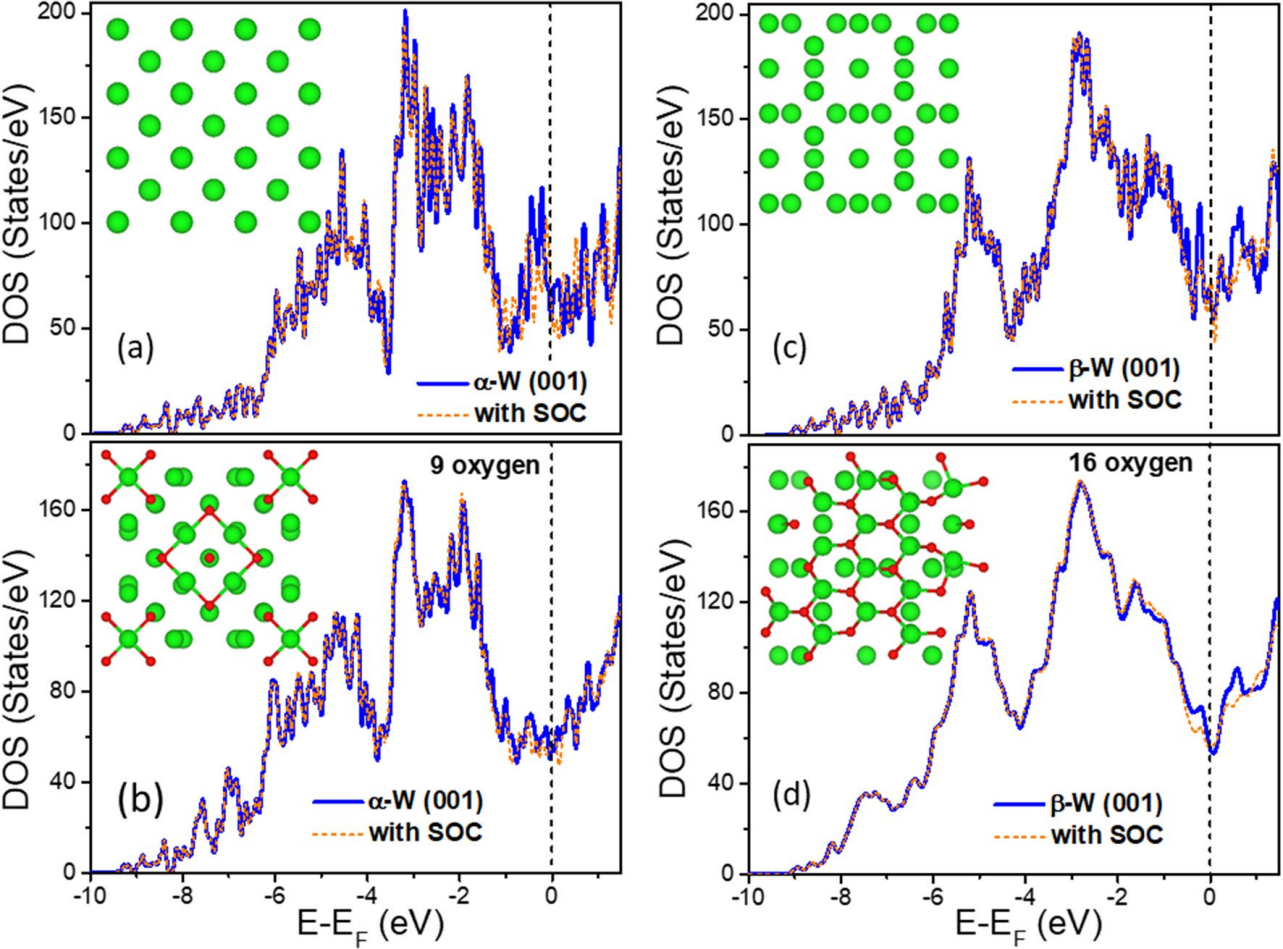
Atomic and electronic structure of (001) slabs of α- and β-W without and with O adsorption. (**a**) and (**c**) TDOS for a slab of α-W and β-W, respectively, with (001) surface (inset shows the top view of the slab in the cell) without and with SOC. (**b**) and (**d**) TDOS of (001) slab of α-W with 9 oxygen on fourfold sites and β-W with 16 oxygen atoms on threefold sites in the supercell, respectively, without and with SOC. The inset shows the top view of the slab in the supercell. Some relaxation of W atoms can be seen particularly for α-W slab. Green (red) balls show W (O) atoms and the vertical broken line shows the Fermi energy E_F_.

We further studied adsorption of O on (001) surfaces of α-W and β-W using a slab. The DOS for the slabs of clean α-W and β-W as well as with O coverage are shown in Fig. 4. We considered adsorption of 9 O (16 O) on one side of the α (β) slab in the supercell. For the α case it corresponds to a p(1 × 1) coverage of oxygen. The optimized atomic structures of the clean and O adsorbed surfaces are shown in the inset. Our results of the DOS for the clean W(001) surface are similar to those reported by Mattheiss and Hamann^31^. The adsorption of oxygen leads to a small displacement of surface W atoms. The DOS of the slab shows a broad peak in the W 5*d* band region. Also, there is a peak at around 7 eV below E_F_ in the DOS of both α and β slabs due to O adsorption as shown in Fig. 4a and b, respectively. It is attributed to O bonding interaction with the slab as it was also found in the case of the O doped bulk β-W [Fig. 3a] and α-W [Fig. 3d]. However, in contrast to the O-doped bulk phases, the DOS is small at around 9 eV below E_F_. These results are not affected much by including SOC. Therefore, the peak at ~ 7 eV below E_F_ can be considered to have the joint contribution of W 5*d* and O 2*p* states from bulk and surface of α and β phases, but the peak around 9 eV below E_F_ will have contributions mainly from bulk α and β phases. These results may vary to some extent depending upon the presence of defects, different surfaces, and/or interfaces such as between α and β phases in actual samples which are not considered in our calculations.

The adsorption energy of an O atom, defined as the gain in energy when a free O atom adsorbs on the surface is 7.50 eV and 7.95 eV, respectively, for a fourfold site of α-W and a threefold site of β-W on a (001) surface. When 9 (16) O atoms are adsorbed on the (001) surface of α-W (β-W) in the supercell, the adsorption energy becomes 6.76 eV (6.74 eV) per O atom. These values are comparable to 6.55 eV and 6.79 eV reported^32^ for (1 × 1) and (2 × 1) structures of O on W(110) surface. It is found that there is a decrease in the adsorption energy of an O atom with increasing coverage. However, the values of the adsorption energy are much higher than the energy gain of 4.031 eV (4.478 eV) per O atom for 12.3 at.% (15.79 at.%) O in a supercell of bulk α-W (β-W). Therefore, O would be there on the surface of both α and β phases, as evidenced from our experiments (see Fig. 2). Further calculations for an O atom in the sub-surface region give only a small (0.223 eV) decrease for α-W but a significantly lower value for β-W. Accordingly, O is likely to be present in sub-surface sites also for α-W, but may go to the bulk region in β-W.

### Effects of O doping on the electronic band structure

In order to further understand the effects of O doping on the band structure near E_F_, we performed calculations on a unit cell of β-W with one and two O atoms. This correspond to 11 at.% and 20 at.% O doping, respectively, covering the range of O concentration of interest. The fully relaxed atomic structure and the unit cell are shown in inset in Fig. S4 in the Supplementary Information. The optimized lattice parameters are 5.122 Å, 5.253 Å, and 5.128 Å (space group Pm) for the case of one O atom and 5.286 Å, 5.286 Å, and 5.069 Å for the doping of 2 O atoms (space group P1). The corresponding DOS are also shown in Fig. S4. These results show O induced peaks in the energy region of − 7.5 eV to − 10 eV and no peak at around − 7 eV. Therefore the latter peak in α- and β-W with higher concentration of O can be associated to arise from clustering of O atoms. There is increase in the DOS at E_F_ with O-doping as we also discussed earlier.

**Figure 5:**
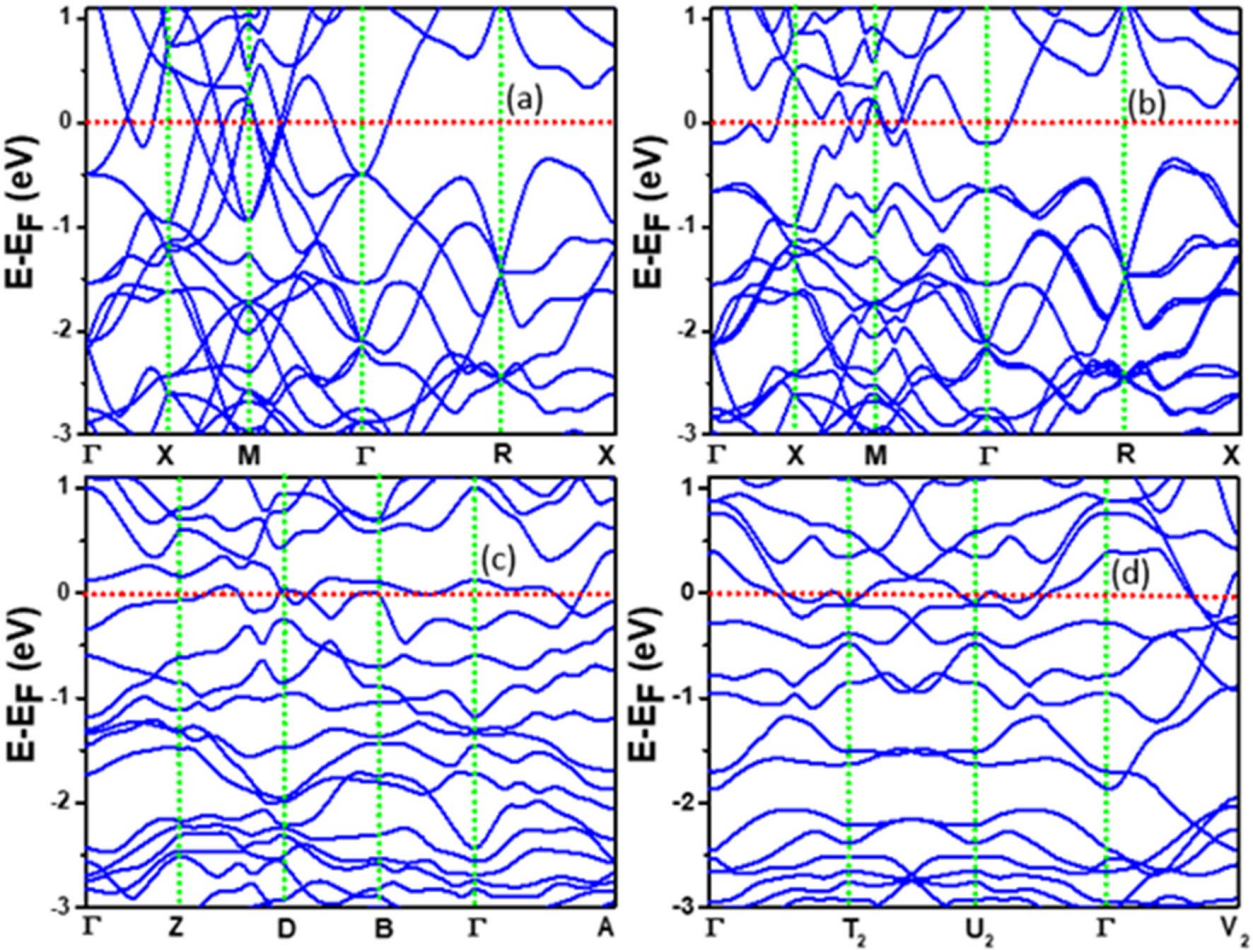
Band structure of pure and O doped β-W. Band structure of pure β-W without (**a**) and with (**b**) SOC. The SOC has significant effect on the energy bands near the Fermi energy which has been taken to be the zero of energy. Band structure for one O (**c**) and two O (**d**) in a unit cell of β-W without SOC. The doping of O creates distortion in the unit cell leading to reduced symmetries with Pm and P1 space groups, respectively.

**Figure 6:**
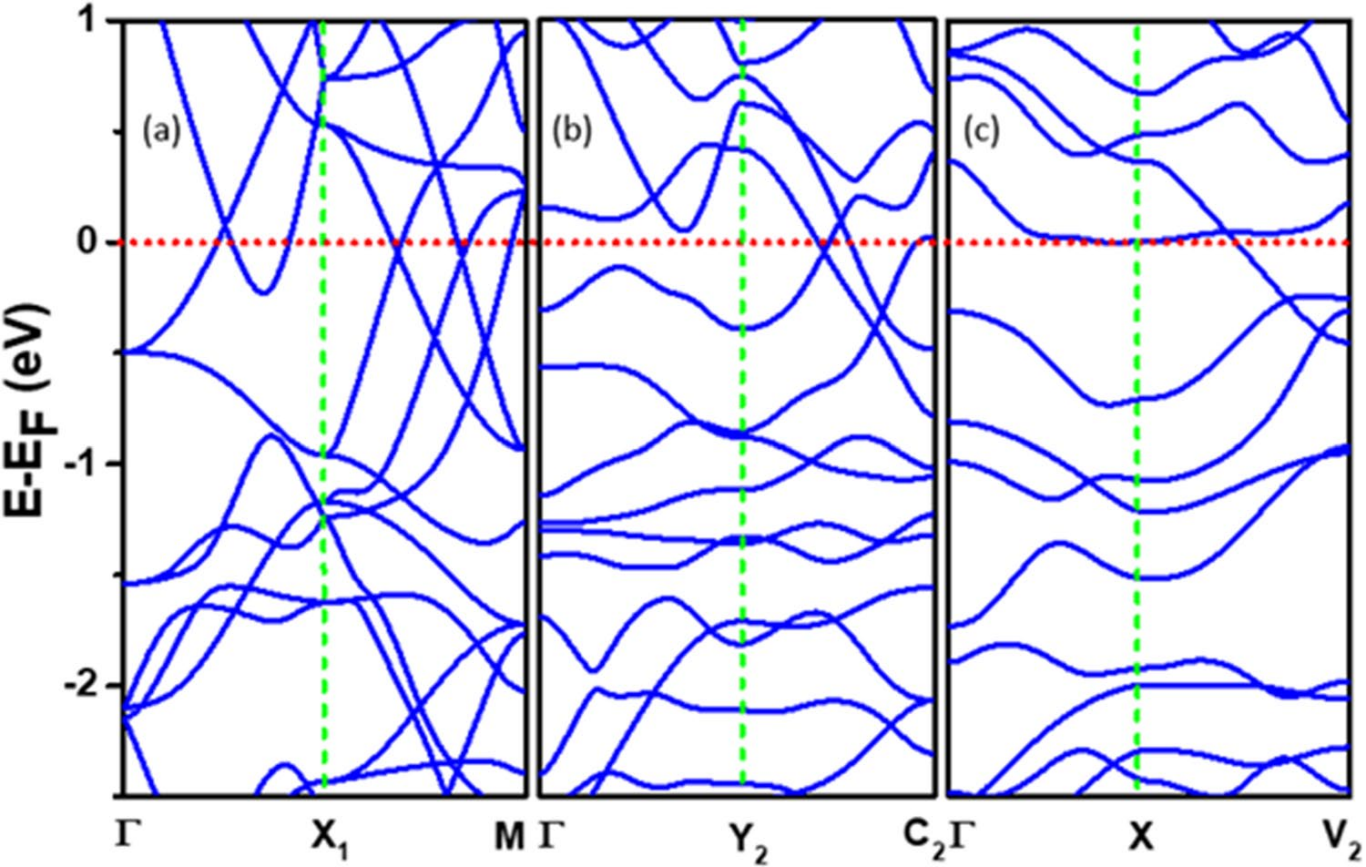
Effects of oxygen doping on energy bands of β-W. (**a**)–(**c**) show the energy bands for the unit cells of undoped, one O doped, and two O doped β-W, respectively, considering nearly equivalent directions in the respective Brillouin zones.

The band structures for the undoped β-W without and with SOC are shown in Fig. 5a and b, respectively. One can see multiple Direc points and nodal lines near E_F_ and this agrees well with the published results^13^. Inclusion of SOC lifts the degeneracy of the bands and opens up small band gaps [Fig. 5b] at some places where the bands cross. Doping of O clearly leads to significant changes in the band structure as it can be seen in Fig. 5c and d for the doping of 1 O and 2 O in the unit cell of β-W, respectively. It has been further found that inclusion of SOC leads to splitting of the bands as shown in Fig. S5 in the Supplementary Information. In order to further compare the results, we have chosen nearly equivalent directions in the Brillouin zones of pure and doped W (see Fig. S6 in Supplementary Information) and the corresponding bands are shown in Fig. 6. Here, the energy bands for the pure β case in Γ-X-M directions [Fig. 6a] having multiple Dirac points near E_F_, get affected when one O is doped [Fig. 6b], but the Dirac point-like feature can still be seen along the Y_2_–C_2_ direction. The bands in the Γ-Y_2_ direction change significantly compared with the Γ-X_1_ direction for the pure case. Further, with the doping of 2 O atoms, the bands have changed more significantly as shown in Fig. 6c. When SOC is included, there is opening of a band gap at the band crossings as shown in Fig. S7. These will contribute to SHA even in the case of O-doped β-W.

### Bonding nature of O and Bader charge analysis

We further explored the nature of bonding by performing Bader charge analysis. It is found that there is ~ 1.6 e excess charge on O atoms predominantly due to charge transfer from the nearest neighbour W atoms. A large fraction of W atoms has charge in the range of 5.5–6.0 e and only a few have charge in the range of 5.0–5.5 e. This suggests that the charge transfer from W to O is much smaller than in WO_3_. The hybridization of partially filled 5*d* orbitals of W with the 2*p* orbitals of O gives rise to an increase in DOS near E_F_ and below ~  − 6 eV (Fig. 3), while a decrease in the remaining *d*-band region of W. The disorder in the structure due to the doping of O also leads to broadening of peaks in the DOS. The isosurfaces of the charge density and electron localization function (ELF) for β-W doped with 15.79 at.% O are shown in Fig. S8. One can see localization of charge around O ions.

## Conclusion

In summary, our ab initio molecular dynamics simulations show that the β phase of W becomes favorable over α with the doping of ~ 20 at.% O. Also our calculations show that oxygen is distributed non-uniformly due to clustering and there could be coexistence of α and β phases. These results are in agreement with our EDX measurements which show about 16.84 at.% O on an average in film B with fluctuation in O concentration. The evolution of stable β phase in W films with O doping was manifested by GIXRD measurements and has been supported by our calculations which also show that O induces disorder in the structure. The electronic structures of O doped bulk β-W as well as α-W show bonding peaks of O 2*p* orbitals with 5*d* orbitals of W at ~ 7 and ~ 9 eV below E_F_ but the peaks in the *d* band region are distinct. There is a characteristic broad peak at around 2.6 eV in the W 5*d* band region of β-W in contrast to peaks at ~ 2 and ~3 eV in pure α-W. There is an increase in DOS at E_F_ in the O-doped β phase with respect to pure α-W. This result is interesting to understand the higher T_c_ in β-W within the BCS formalism. Oxygen is found to interact strongly on W surfaces and therefore it supports the observed increase in O concentration in the surface region. These results would be interesting for further experimental and theoretical studies of the surfaces and interfaces of β-W films and their frontier applications. The presence of O induced disorder in the structure as well as its inhomegenous distribution will contribute to the higher resistivity of β-W as observed compared with a pure BCC phase due to increased scattering of electrons. But real samples are polycrystalline with small grain size and a proper understanding will need such consideration including the role of defects and interfaces. We believe that our combined experimental and theoretical study will help in further understanding of the technologically interesting β phase of W.

## Methods

### Sample preparation and measurements

A 500 µm thick Si(100) wafer was diced into pieces (area 1 × 1 cm^2^) and cleaned by dipping in trichloroethylene, acetone, isopropanol, and deionized water followed by heating for 2 min in each step. After cleaning of the Si surface, W films of about 35 nm (film A) and 60 nm (film B) thicknesses were grown at room temperature (RT) by electron beam deposition technique. The deposition rate was 0.01 nm/s, while the chamber base pressure and the working pressures were ~ 6.65 × 10^–7^ mbar and 1.33 × 10^–6^ mbar, respectively. We used 99.95% pure W powder (Alfa Aesar) for depositing films whose thickness was estimated by a surface profilometer (DektakXT, Bruker). The β-W phase was confirmed by GIXRD measurements (Bruker, D8-Discover) using the Cu-*K*_*α*_ radiation (*λ* = 0.154 nm). The elemental analysis has been performed by EDX with a 2 nm spot size in the cross-sectional geometry of TEM. The mapping has been taken parallel to the film surface. We used a C_s_ corrected TEM system from Tecnai-FEI operated with an acceleration voltage of 200 kV. A Pt layer was deposited on the W film to avoid any damage during the XTEM sample preparation by focused ion beam. The full range of the EDX hypermap has 172 (69) values in a column for each element in every raster scan for sample B (A).

### Calculations on bulk and surfaces

The calculations have been performed within the framework of DFT as implemented in the Vienna Ab initio Simulation Package (VASP)^33^. We used Perdew-Burke-Ernzerhof (PBE) form of the generalized gradient approximation (GGA)^34^ for the exchange–correlation functional and projected augmented wave (PAW) pseudopotentials^35^ for electron–ion interaction. The kinetic energy cut-off for the plane wave expansion of the wave function was set to 500 eV. The atomic structures in all cases were completely relaxed until the absolute value of each component of the Hellmann–Feynman force on each ion became less than 0.005 eV/Å. We also performed the volume relaxation. A grid of 15 × 15 × 15 (9 × 9 × 9) Monkhorst–Pack **k**-points was used for the unit cell calculations of the α (β) phase, whereas 5 × 5 × 5 **k**-points were used for the 4 × 4 × 4 (2 × 2 × 2) supercell calculations for structural relaxation and the convergence of the charge density. The lattice parameters of the optimized unit cells of 1) α-W (bcc, Im-3 m) with 2 atoms per unit cell and 2) β-W (A15, Pm-3n) containing 8 atoms per unit cell, are found to be 3.171 Å and 5.056 Å, respectively. These are in good agreement with the previously reported values of 3.17 Å and 5.05 Å as well as our experimental results^1,2,4,18^. For the 2 × 2 × 2 supercell of β-W phase (64 atoms) the optimized lattice parameter was found to be 10.110 Å. Oxygen with 15.79 at.% concentration was initially distributed in β-W supercell keeping the O–O separation of about 2.04 Å as obtained for the case of two O atoms. This oxygen concentration lies in the range of 13–19% obtained from the EDX measurement^18^ on sample B. Subsequently we performed ab initio MD simulations to explore energetically the most favorable configuration using the simulated annealing method. For this, the system was heated to 3500 K and equilibrated for 3 ps. Then the system was cooled from 3500K to room temperature continuously by simulating for 3.5 ps. In these finite temperature calculations, we used only the Γ point to represent the Brillouin zone. After cooling to room temperature, we optimized the structure by relaxing the ions as well as the cell parameters using again 5 × 5 × 5 **k**-points mesh with high precision. Similar calculations were performed for other oxygen concentrations as well as for α-W. The (001) surface of β-W was modelled by a slab of 3 unit-cell thickness (108 W atoms) with about 15 Å vacuum space. We considered 2 × 2 (3 × 3) supercell in the plane of the slab for β-W (α-W with 99 atoms) and a 5 × 5 × 1 **k**-points mesh to perform Brillouin zone integrations. The ions were relaxed keeping the cell dimensions fixed. Further we studied adsorption of O atoms at fourfold (threefold) site on the (001) surface of α (β) as well as on a sub-surface site in order to compare the behavior with bulk. Bader charge analysis has been conducted to check the valence electrons on W and O atoms.

## Supplementary Information

Supplementary Information.

## References

[1] Demasius KU, Phung T, Zhang W, Hughes BP, Yang SH, Kellock A, Han W, Pushp A, Parkin SS (2016). Enhanced spin–orbit torques by oxygen incorporation in tungsten films. Nat. Comm..

[2] Pai CF, Liu L, Li Y, Tseng HW, Ralph DC, Buhrman RA (2012). Spin transfer torque devices utilizing the giant spin Hall effect of tungsten. Appl. Phys. Lett..

[3] Hao Q, Xiao G (2015). Giant spin Hall effect and switching induced by spin-transfer torque in a W/Co_40_Fe_40_B_20_/MgO structure with perpendicular magnetic anisotropy. Phys. Rev. Appl..

[4] Sui X, Wang C, Kim J, Wang J, Rhim SH, Duan W, Kioussis N (2017). Giant enhancement of the intrinsic spin Hall conductivity in β-tungsten via substitutional doping. Phys. Rev. B.

[5] Costa M, Costa AT, Hu J, Wu RQ, Muniz RB (2018). *β*-tungsten: a promising metal for spintronics. J. Phys. Condens. Matter.

[6] Sethu KKV, Ghosh S, Couet S, Swerts J, Sorée B, Boeck JD, Kar GS, Garello K (2021). Optimization of Tungsten β-phase window for spin-orbit-torque magnetic random-access memory. Phys. Rev. Appl..

[7] Davey WP (1925). The lattice parameter and density of pure tungsten. Phys. Rev..

[8] Charlton MG, Davis GL (1955). Allotropes of tungsten. Nature.

[9] Petroff P, Sheng TT, Sinha AK, Rozgonyi GA, Alexander FB (1973). Microstructure, growth, resistivity, and stresses in thin tungsten films deposited by rf sputtering. J. Appl. Phys..

[10] O’Keefe MJ, Grant JT (1996). Phase transformation of sputter deposited tungsten thin films with A-15 structure. J. Appl. Phys..

[11] Rossnagel SM, Noyan IC, Cabral C (2002). Phase transformation of thin sputter-deposited tungsten films at room temperature. J. Vac. Sci. Technol. B.

[12] Choi D (2017). Phase transformation in thin tungsten films during sputter deposition. Microelec. Eng..

[13] Li J, Ullah S, Li R, Liu M, Cao H, Li D, Li Y, Chen XQ (2019). Topological massive Dirac fermions in β-tungsten. Phys. Rev. B.

[14] Shen YG, Mai YW, Zhang QC, McKenzie DR, McFall WD, McBride WE (2000). Residual stress, microstructure, and structure of tungsten thin films deposited by magnetron sputtering. J. Appl. Phys..

[15] Shen YG, Mai YW (2000). Influences of oxygen on the formation and stability of A15 β-W thin films. Mater. Sci. Eng. A.

[16] Weerasekera IA, Shah SI, Baxter DV, Unruh KM (1994). Structure and stability of sputter deposited beta-tungsten thin film. Appl. Phys. Lett..

[17] Narasimham AJ, Medikonda M, Matsubayashi A, Khare P, Chong H, Matyi RJ, Diebold A, LaBella VP (2014). Fabrication of 5–20 nm thick β-W films. AIP Adv..

[18] Chattaraj A, Balal M, Yadav AK, Barman SR, Sinha AK, Jha SN, Joulie S, Serin V, Claverie A, Kumar V, Kanjilal A (2020). Unravelling oxygen driven α to β phase transformation in tungsten. Sci. Rep..

[19] Qiu X, Narayanapillai K, Wu Y, Deorani P, Yang DH, Noh WS, Park JH, Lee KJ, Lee HW, Yang H (2015). Spin–orbit-torque engineering via oxygen manipulation. Nat. Nanotech..

[20] Chopra KL (1967). Enhancement of superconductivity in tungsten films. Phys. Lett. A.

[21] Basavaiah S, Pollack SR (1968). Superconductivity in β-Tungsten Films. J. Appl. Phys..

[22] Lita AE, Rosenberg D, Nam S, Miller AJ, Balzar D, Kaatz LM, Schwall RE (2005). Tuning of tungsten thin film superconducting transition temperature for fabrication of photon number resolving detectors. IEEE Trans. Appl. Supercond..

[23] Bond WL, Cooper AS, Andres K, Hull GW, Geballe TH, Matthias BT (1965). Superconductivity in films of β tungsten and other metals. Phys. Rev. Lett..

[24] Young BA, Saab T, Cabrera B, Cross JJ, Abusaidi RA (2000). Tc tuning of tungsten transition edge sensors using iron implantation. Nucl. Instr. Meth. Phys. Res. A.

[25] Mata-Pinzón Z, Valladares AA, Valladares RM, Valladares A (2016). Superconductivity in bismuth. A new look at an old problem. PLoS ONE.

[26] Hao Q, Chen W, Xiao G (2015). Beta (β) tungsten thin films: Structure, electron transport, and giant spin Hall effect. Appl. Phys. Lett..

[27] Derunova E, Sun Y, Felser C, Parkin SSP, Yan B, Ali MN (2019). Giant intrinsic spin Hall effect in W_3_Ta and other A15 superconductors. Sci. Adv..

[28] Minhas MZ, Pandeya AK, Grover B, Fumarola A, Kostanovskiy I, Hazra BK, Hoppe W, Woltersdorf G, Bedoya-Pinto A, Parkin SSP, Ali MN (2020). Doping-induced spin Hall ratio enhancement in A15-phase, Ta-doped β-W thin films. J. Phys. Mater..

[29] Qian L, Wang K, Zheng Y, Xiao G (2020). Spin Hall effect in the α and β phases of Ta_x_W_1-x_ alloys. Phys. Rev. B.

[30] Jansen HJF, Freeman AJ (1984). Total-energy full-potential linearized augmented-plane-wave method for bulk solids: Electronic and structural properties of tungsten. Phys. Rev. B.

[31] Mattheiss LF, Hamann DR (1984). Electronic structure of the tungsten (001) surface. Phys. Rev. B.

[32] Petrova NV, Yakovkin IN (2009). Binding energies for oxygen on transition metal surfaces. Surf. Rev. Lett..

[33] Kresse G, Joubert D (1999). From ultrasoft pseudopotentials to the projector augmented-wave method. Phys. Rev. B.

[34] Perdew JP, Burke K, Ernzerhof M (1996). Generalized gradient approximation made simple. Phys. Rev. Lett..

[35] Blöchl PE (1994). Projector augmented-wave method. Phys. Rev. B.

