# Supplementary material for: Crucial role of oxygen on the bulk and surface electronic properties of stable β phase of tungsten
Source: Sci Rep. 2022 Mar 9;12:3865. doi: 10.1038/s41598-022-07658-7 (PMC8907277; doi:10.1038/s41598-022-07658-7)
Supplement: Supplementary file 1 — Supplementary Information. [file 41598_2022_7658_MOESM1_ESM.pdf]

SUPPLEMENTARY INFORMATION

Crucial role of oxygen on the bulk and surface electronic properties of stable β phase of tungsten

Ananya Chattaraj[1], Sebastien Joulie[2], Virginie Serin[2], Alain Claverie[2], Vijay Kumar[3,4]* and Aloke Kanjilal[1]*

[1]*Department of Physics, School of Natural Sciences, Shiv Nadar University, NH-91, Tehsil Dadri, Gautam Buddha Nagar, Uttar Pradesh 201 314, India*

[2]*CEMES-CNRS and Université de Toulouse, 29 rue J. Marvig, 31055 Toulouse, France*

[3]*Center for Informatics, School of Natural Sciences, Shiv Nadar University, NH91, Tehsil Dadri, Gautam Buddha Nagar, Uttar Pradesh 201 314, India*

[4]*Dr. Vijay Kumar Foundation, 1969 Sector 4, Gurgaon, Haryana 122001, India*

*Corresponding authors: vijay.kumar@snu.edu.in (VK), aloke.kanjilal@snu.edu.in (AK)

TABLE S1. Calculated lattice parameters and energy gain due to O doping for different oxygen concentrations in bulk β-W. For pure β (A15), the lattice parameter is for a unit cell whereas for the O doped cases, we used a 2×2×2 supercell.

Oxygen concentration	Lattice parameters (Å)	Energy gain (eV/O)
0	5.056 (A15)	
20 at.%	10.635, 10.638, 10.588	4.720
27.27 at.%	10.993, 10.669, 10.671	4.793
30.43 at.%	10.988, 10.670, 10.671	4.859

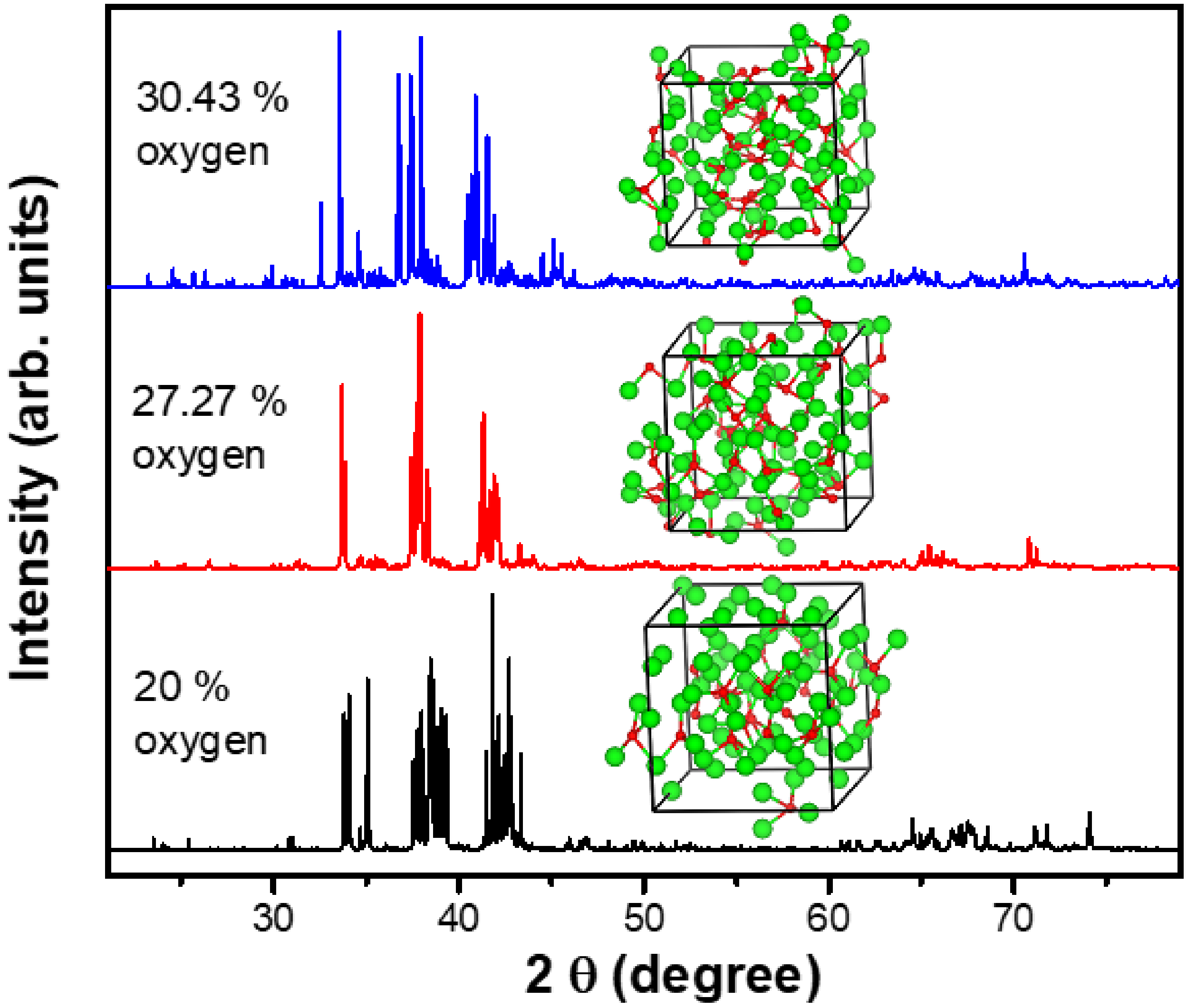

Figure S1 Calculated powder X-ray diffraction pattern of O-doped β-W. The powder X-ray diffraction pattern of simulated β-W doped with 20 at.%, 27.27 at.%, and 30.43 at.% oxygen concentrations. The corresponding atomic structures obtained from *ab initio* molecular dynamics simulations are shown in inset. The higher oxygen concentration leads to increasing disorder in the structure and increase in the lattice parameters. Green (red) balls show W (O) atoms. Clustering of O atoms can be seen.

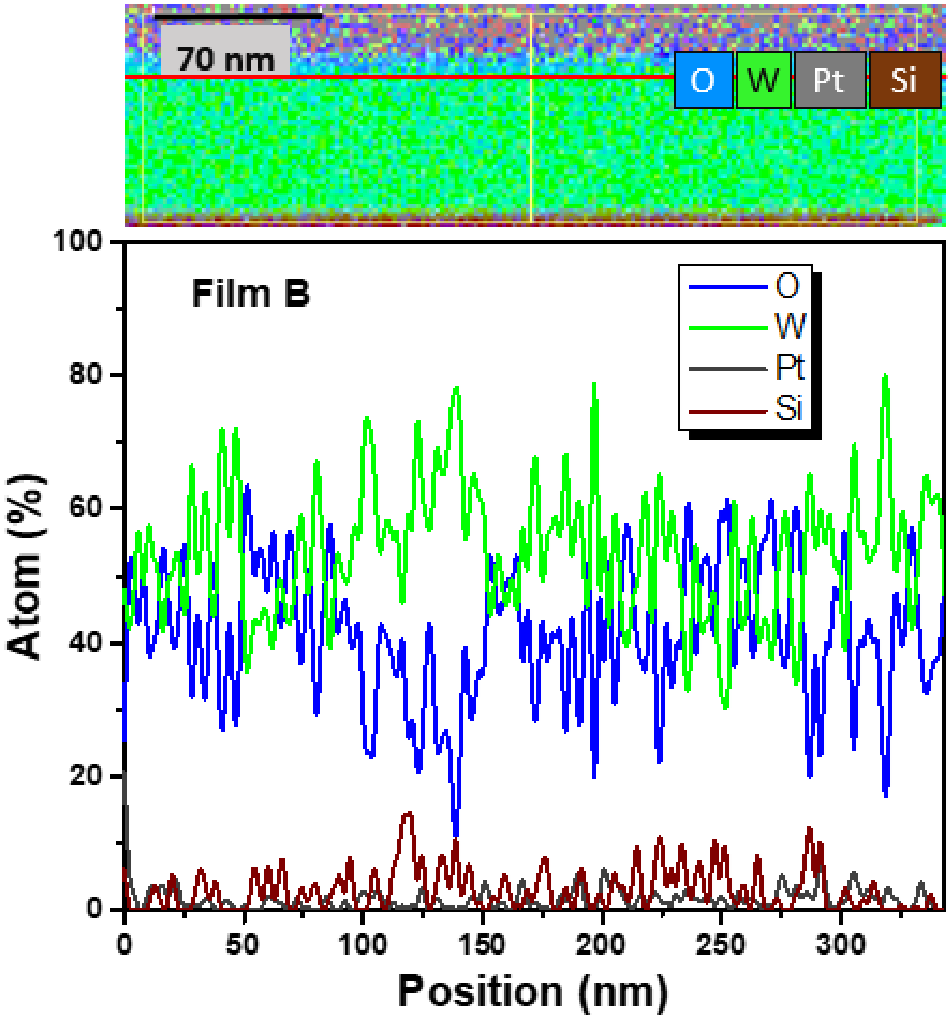

Figure S2 Elemental distribution in the surface region of 60 nm thick film B by EDX. The superimposed elemental maps by EDX show the distribution of W (green), Si (brown), O (blue) and Pt (gray) near the surface of the W film (trace marked by red line) during XTEM investigations. The elemental profiles along the trace parallel to the surface in the top panel, are projected in the below panel. There is much increase in the average oxygen concentration to 41.48 at.% with a standard deviation of 14.31 at.%. The Si and Pt signals remain below the noise level.

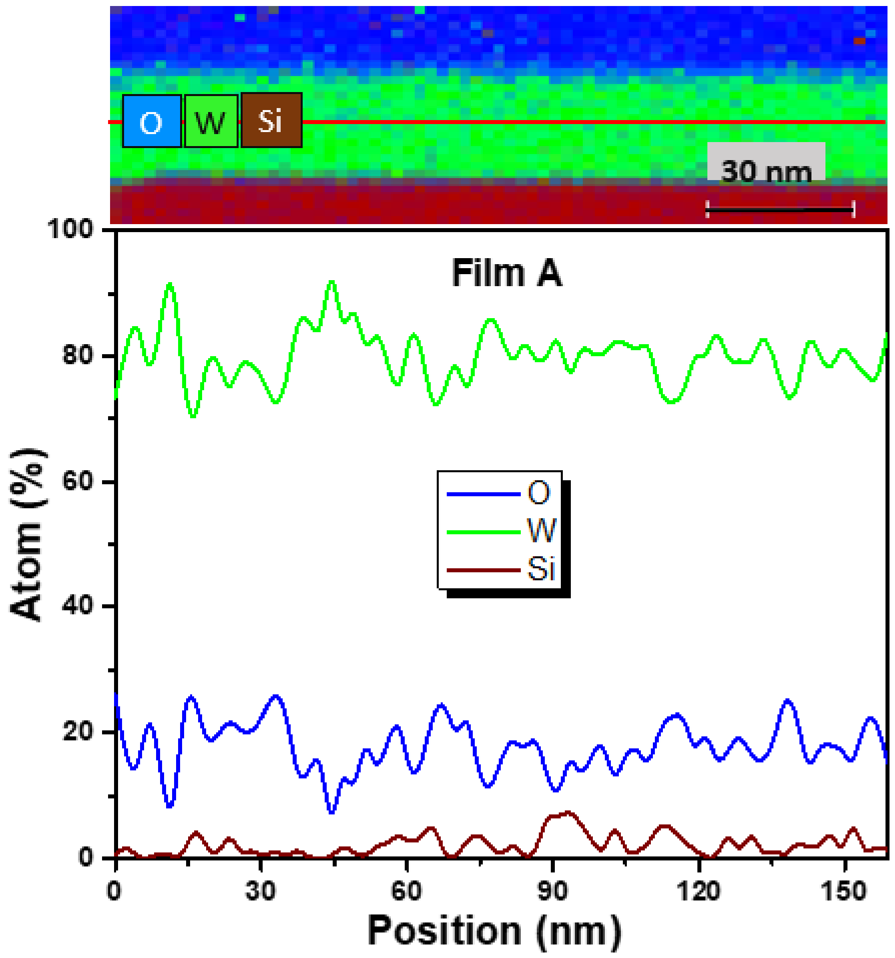

Figure S3 Elemental distribution in 35 nm thick film A by EDX. The superimposed EDX maps (69 × 28 pixels) show the elemental distribution of W (green), Si (brown), and O (blue). The elemental profiles along the trace around the middle of the film (red line), parallel to the surface in the top panel, is projected in the lower panel showing the average oxygen concentration of 17.75 at.% with a standard deviations of 5.27 at.%. The Si signal in this region is below the noise level.

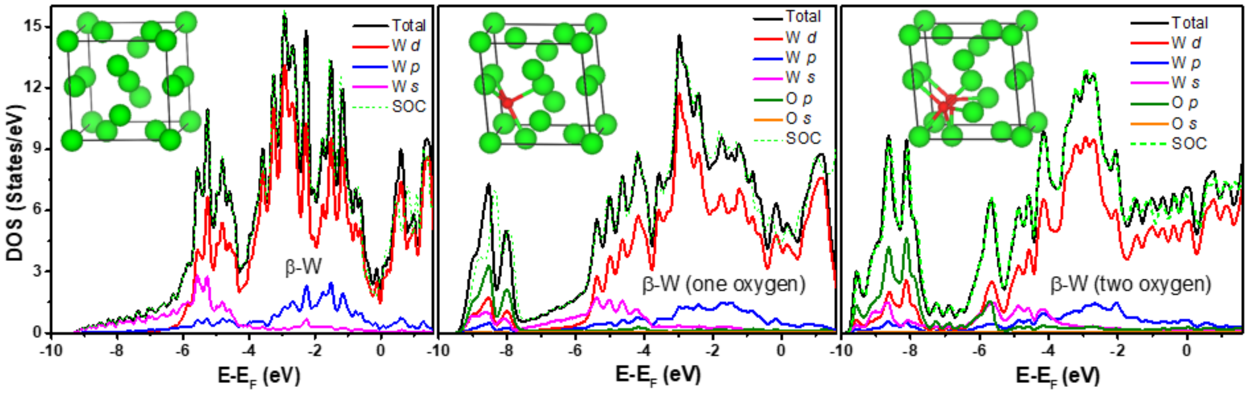

Figure S4 Electronic density of states for pure and O doped β-W. TDOS and angular momentum as well as site projected DOS for a unit cell of pure β-W, with one O, and two O in the unit cell without using spin-orbit coupling (SOC). TDOS with SOC is also shown and there is only a small change. Green (red) balls show W (O) atoms.

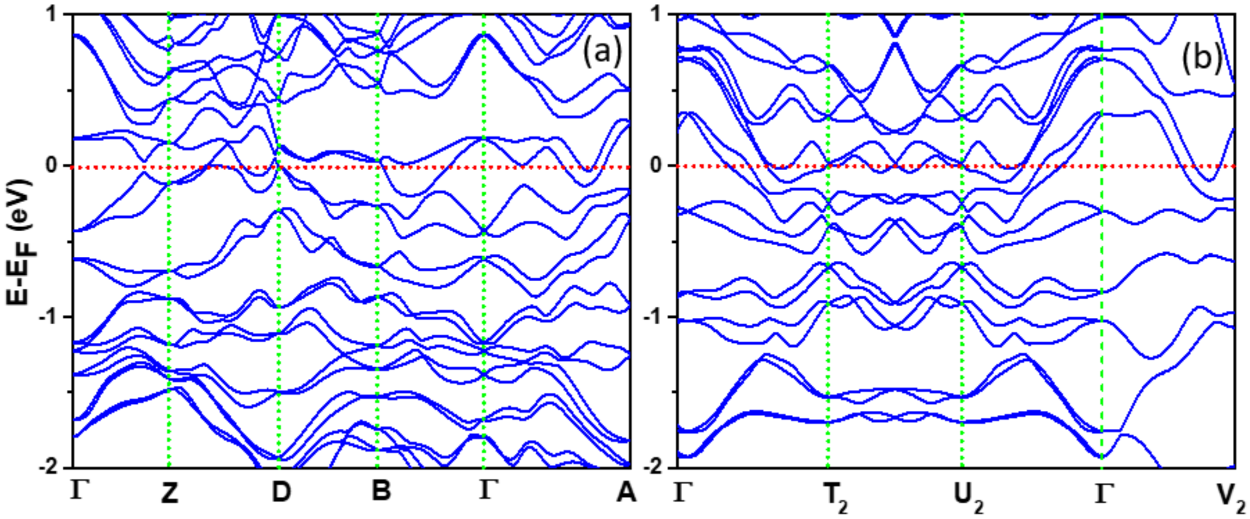

Figure S5 Effects of spin-orbit coupling on the energy bands of O-doped β-W. The energy bands for (a) one O and (b) two O in a unit cell of β-W after including SOC. The splitting of energy bands can be seen.

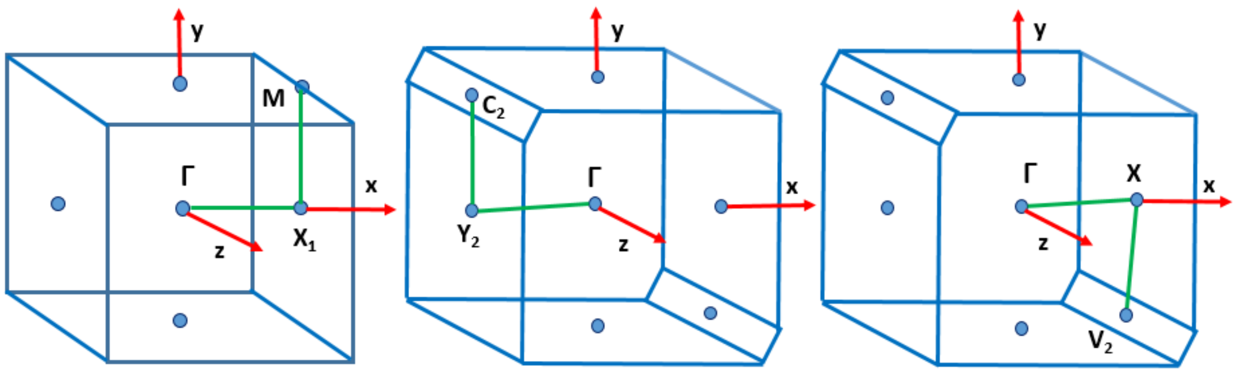

Figure S6 Brillouin zones for pure and O doped unit cells. Symmetry directions in the Brillouin zones for (left to right) pure β-W, with one O in the unit cell (space group Pm), and with two O in the unit cell (space group P1).

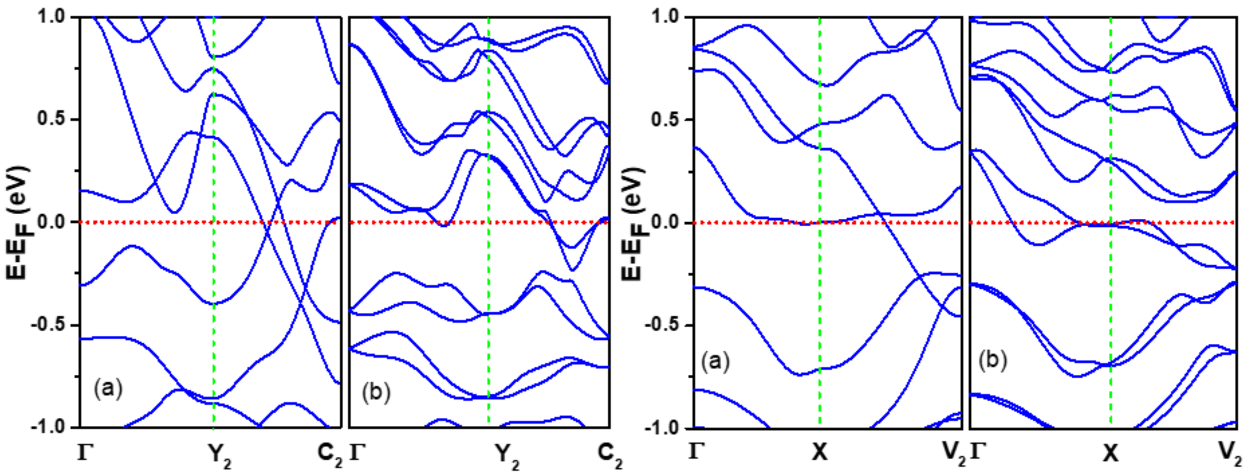

Figure S7 Effects of spin-orbit coupling on the energy bands in nearly equivalent directions. The energy bands for (left panels) one O and (right panels) two O in the unit cell of β-W. In each case (a) and (b) correspond to bands without and with inclusion of SOC, respectively. The opening of a band gap as well as splitting of the bands can be seen.

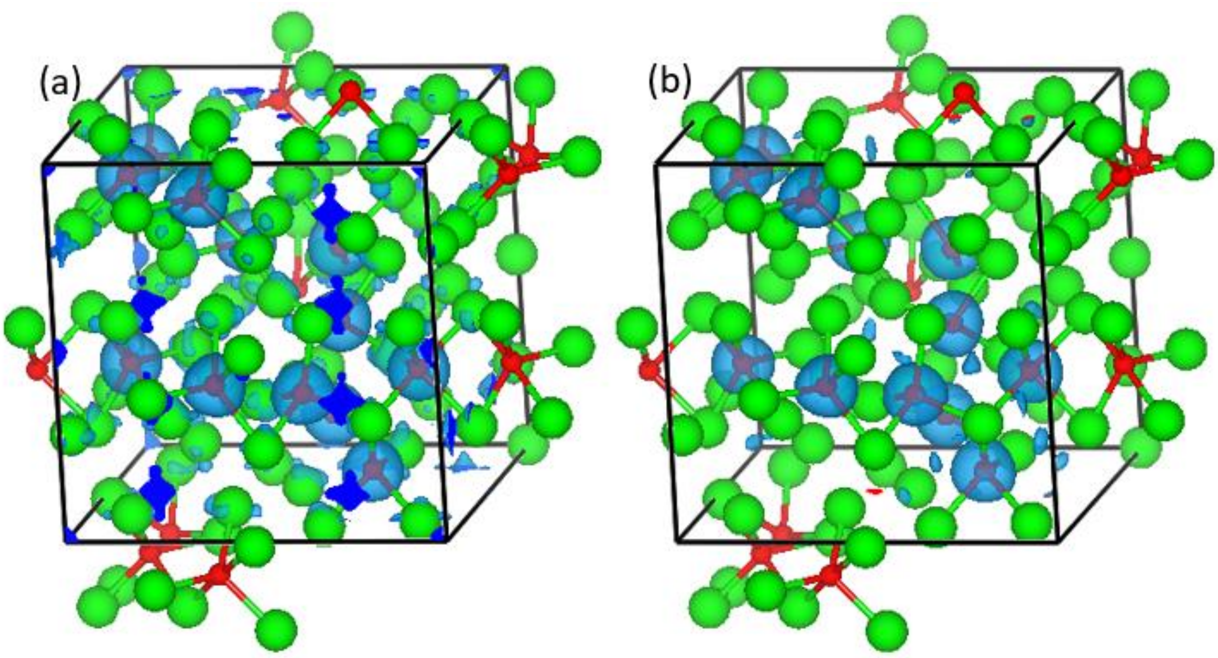

Figure S8 Isosurfaces of charge density and ELF. Isosurfaces of (a) electronic charge density and (b) ELF for β-W doped with 15.79 at.% O. The isosurface values are 0.1 e/$Å^3$ and 0.6, respectively. It can be seen that the charge is localized around the O ions.
